# Dietary Patterns Influence Target Gene Expression through Emerging Epigenetic Mechanisms in Nonalcoholic Fatty Liver Disease

**DOI:** 10.3390/biomedicines9091256

**Published:** 2021-09-18

**Authors:** Mohamed Zaiou, Rim Amrani, Bertrand Rihn, Tahar Hajri

**Affiliations:** 1 The Jean-Lamour Institute, UMR 7198 CNRS, University of Lorraine, F-54000 Nancy, France; bertrand.rihn@univ-lorraine.fr; 2 Department of Neonatology, University Mohammed First, Oujda 60000, Morocco; r.amrani@ump.ac.ma; 3 Department of Human Ecology, Delaware State University, Dover, DE 1191, USA; thajri@desu.edu

**Keywords:** epigenome, nonalcoholic fatty liver disease (NAFLD), metabolic associated fatty liver disease (MAFLD), nutrition, DNA methylation, histone modifications, gene expression, hepatic steatosis

## Abstract

Nonalcoholic fatty liver disease (NAFLD) refers to the pathologic buildup of extra fat in the form of triglycerides in liver cells without excessive alcohol intake. NAFLD became the most common cause of chronic liver disease that is tightly associated with key aspects of metabolic disorders, including insulin resistance, obesity, diabetes, and metabolic syndrome. It is generally accepted that multiple mechanisms and pathways are involved in the pathogenesis of NAFLD. Heredity, sedentary lifestyle, westernized high sugar saturated fat diet, metabolic derangements, and gut microbiota, all may interact on a on genetically susceptible individual to cause the disease initiation and progression. While there is an unquestionable role for gene-diet interaction in the etiopathogenesis of NAFLD, it is increasingly apparent that epigenetic processes can orchestrate many aspects of this interaction and provide additional mechanistic insight. Exciting research demonstrated that epigenetic alterations in chromatin can influence gene expression chiefly at the transcriptional level in response to unbalanced diet, and therefore predispose an individual to NAFLD. Thus, further discoveries into molecular epigenetic mechanisms underlying the link between nutrition and aberrant hepatic gene expression can yield new insights into the pathogenesis of NAFLD, and allow innovative epigenetic-based strategies for its early prevention and targeted therapies. Herein, we outline the current knowledge of the interactive role of a high-fat high-calories diet and gene expression through DNA methylation and histone modifications on the pathogenesis of NAFLD. We also provide perspectives on the advancement of the epigenomics in the field and possible shortcomings and limitations ahead.

## 1. Introduction

Nonalcoholic fatty liver disease (NAFLD) includes a spectrum of features spanning from the simple accumulation of triglycerides (TG) in hepatocytes (hepatic steatosis) to nonalcoholic steatohepatitis (NASH), which is characterized by the presence of an inflammatory infiltrate and hepatocellular injury [1], and may further evolve to cirrhosis and hepatocellular carcinoma (HCC) [2]. Based on the close association between hepatic steatosis and metabolic dysregulation, international consensus guidelines recommended the renaming of NAFLD to metabolic associated fatty liver disease (MAFLD) [3,4]. Several emerging research studies are providing support for the shift to the novel nomenclature and its criteria for diagnosis. For example, van Kleef et al. suggested recently that using novel MAFLD criteria would help to improve the identification and treatment of fatty liver disease patients at risk for fibrosis [5]. Others investigations demonstrated the importance of MAFLD criteria in identifying individuals with impaired liver health and increased cardiovascular risk [6,7,8]. However, the proposed terminology may change with substantial advancement of our scientific knowledge in the field.

NAFLD is emerging as the most common cause of chronic liver disease, especially in countries that consume a western diet that is high in saturated fat, trans fat, and refined sugars [9]. The prevalence of NAFLD was estimated to be between 25–45% of the general population [10], and 70–90% among patients with metabolic comorbidities such as obesity, type 2 diabetes mellitus (T2DM) or metabolic syndrome (MetS) [11]. In fact, epidemiological and clinical studies demonstrated that NAFLD has, in addition to intrahepatic lesions, devastating health consequences beyond the liver, and is commonly intimately linked to metabolic disorders such as obesity, insulin resistance, T2DM [12,13], inflammation, mitochondrial damage, and oxidative stress response [14]. In addition, patients with NAFLD are at substantial risk for the development of cardiovascular diseases (CVD) [15]. Since NAFLD is recognized as the hepatic manifestation of metabolic syndrome, a recent study suggested that the inclusion of steatosis in the panel of MetS diagnostic risk factors improves the predictive power of cardiovascular risk better than the current MetS criteria [16].

**Figure 1:**
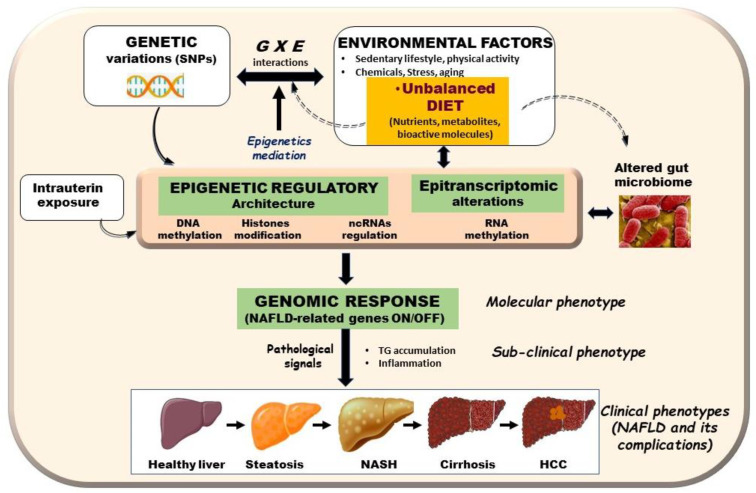
Schematic representation of potential epigenetic and genetic events that are altered by diet leading to NAFLD-related genes aberrant expression. Environment factors (*E*) such as nutrition, genetics factors (*G*), gut microbiota, and intrauterine environment, act collectively in play with epigenetic landscape to induce NAFLD phenotype and associated complications. Diet represents one of greatest environmental determinants of an individual’s health. Nutrients, metabolites, and bioactive components can reversibly alter epigenetics marks causing epigenetics alterations in known epigenetic mechanisms: DNA methylation, histone modifications, noncoding RNAs regulation, and most likely, RNA epigenetics. Resulting epigenetic alterations impact genome by affecting metabolic gene expression patterns, and accordingly, lead to metabolic diseases such as NAFLD. These events highlight role of epigenetic alterations as interface between *E* (e.g., diet/metabolism) and *G* (e.g., genetic variations) interactions in metabolic disorders including NAFLD.

The root causes of NAFLD were extensively debated during the last few years. While investigations brought forward evidence that this disorder may be caused by a plethora of modifiers including sedentary lifestyle, metabolic derangements, gut microbiota, genetic predisposition, and epigenetic factors [1], unhealthy diet remains the factor that contributes the most (Figure 1) [17]. With respect to genetic component, studies of families and twins as well as genome wide association studies (GWAS) provided evidence for an element of heritability in NAFLD [18,19]. GWAS carried out mainly in adult cohorts led to the identification of various genetic variants that potentially could serve as biomarkers for early prediction of individual risks [20]. Among these, genetic variants in patatin-like phospholipase domain containing three protein (*PNPLA3*), transmembrane 6 superfamily member 2 (*TM6SF2*), and membrane bound O-acyltransferase domain-containing seven gene (*MBOAT7*), which are involved in lipid droplets remodeling and very low-density lipoproteins secretion, are considered as the major determinants of interindividual differences in NAFLD trait [21,22]. However, the specificity of these variants remains unknown and genetics alone cannot explain the large variability in the prevalence of NAFLD [23].

Environmental factors including sedentary lifestyle, overconsumption of a high-fat western type diet (HFD), and increased intake of sweetened beverages are major risk factors for the onset and progression of NAFLD [24]. In this respect, a recent report indicated that a HFD-induced maternal hypercholesterolemia predisposed offspring to NAFLD and metabolic diseases [25]. Moreover, overwhelming studies established a direct link between various nutrients consumption and long-term liver damage from NAFLD [26,27]. In a similar way, Nobili et al. reported an association between fructose consumption and NASH in a cohort of children and adolescents with a histologically confirmed diagnosis of NAFLD [28]. Conversely, the restriction of fructose intake was associated with a reduction in hepatic fat content and *de novo* lipogenesis [29]. More interesting, human data suggest that exposure to excess maternal fuels during pregnancy could prime the fetal liver for NAFLD and might drive the risk for NASH in the next generation [30]. Together, these findings imply that healthful dietary patterns and the intake of unsaturated fats are protective against NAFLD. In addition, certain dietary supplements could be useful in preventing the development and/or worsening of liver steatosis in patients with NAFLD. Nowadays, healthy diet habit represents a key factor to enhance the health status and welfare; indeed, within the scientific community, diet supplementation was widely accepted as useful strategy to modulate and/or optimize the biochemical and molecular pathways which orchestrate the metabolic responses to both physiological and pathological conditions [31]. Of note, since dietary habits and lifestyle play a chief role in the prevention and treatment of NAFLD in humans, the search for effective nutritional strategy to reduce risk of liver disease is worthy of investigation [32].

Although genetic and environmental factors were thought to be independently associated with metabolic disorders, huge evidence confirms the existence of complex interactions between genetic background and environmental influences, particularly diet, to modulate individual risk of NAFLD development and its severity and progression [33]. This is not surprising, since nutritional genomic studies revealed that nutrition is most likely the key environmental factor that exerts its impact on health outcomes by directly affecting the expression of key genes involved in major metabolic pathways. Moreover, nutrigenetics provided evidence that genetic variants can be associated with differential response to nutrients and affect health outcomes relating this variation to variable disease states. However, how the bidirectional interaction between nutrition and an individual’s genetic makeup impacts health status is not well-understood. In this respect, progress in the field may come from the emerging knowledge of nutriepigenomics, referred to as the interaction between nutrients and genome through epigenetic mechanisms.

Epigenetics was originally defined as heritable changes in gene expression without altering the primary DNA sequence [34]. While the genome is identical in all cells of an organism, the epigenome contains key information specific to every type of cells. Modulation of gene expression can occur through the epigenetic landscape or epigenome, a complex network of modifications including DNA methylation, histone protein posttranslational modifications, chromatin remodeling, and several noncoding RNAs (ncRNAs) regulation [4,35]. These dynamic processes may be responsible for mediating gene–gene and gene–environmental interactions, which consequently induce phenotypic changes. Indeed, multiple studies suggested that epigenetic factors may contribute to the metabolic memory in liver tissue [36]. Thus, attempts were made to identify epigenetic mechanisms underlying metabolic alterations caused by diet-induced NAFLD, as these could be beneficial for disease treatment. Specifically, the effect of HFD on genes involved in hepatic fat accumulation and steatosis was shown to be mediated by epigenetic factors, which play crucial roles in the molecular initiation of liver dysfunction and NAFLD development [37,38]. Despite these encouraging data, we still do not have a firm handle on how and when epigenetic marks that occur in response to an HFD alter gene expression in NAFLD. To fill this knowledge gap, there is clearly a need for a streamlined and novel investigation of epigenetic machinery that interacts with master regulators of lipogenic and glycolytic gene expression programs. Understanding this potential interaction and the resulting pathological signals may lead to identification of epigenetic marks that predispose an individual to NAFLD, and subsequently, could allow early preventive and therapeutic strategies for those at a high risk for the disease.

## 2. Epigenetic Mechanisms Underlying the Link between Nutrition and Aberrant Gene Expression in NAFLD

As discussed above, NAFLD susceptibility and progression are likely attributed to dynamic interactions between genetic and environmental factors [18,39]. However, knowledge surrounding molecular mechanisms by which these factors, particularly diet, alter hepatic gene expression to trigger NAFLD remains limited. A large body of evidence strongly supports that alteration in epigenetic landscape mediates gene–diet interaction and play important roles in the onset of the NAFLD [18]. The major elements of the human epigenome are covalent chemical changes to DNA and histones that contribute to the fine-tune regulation of gene expression and changes of chromatin structure [4]. But the question is: how can HFD connect metabolic information with transcriptional gene control through epigenetics marks to initiate NAFLD? Preliminary observations suggest that biochemical modifications to DNA and certain histones involve several modifying enzymes that play important roles in epigenetic gene regulation. The activity of these enzymes is sensitive to dietary factors and cofactors generated by cellular intermediary metabolism, allowing cells to adapt to a change in conditions by switching specific genes on and off, thereby providing a link between diet, metabolism, and gene expression [40]. As an example, metabolites deriving from various food sources can serve as substrates or cofactors for transcription factors histone modifying enzymes that affect chromatin compaction, leading to transcriptional regulation associated with diseases and ageing [41].

In addition to DNA methylation and histones modifications, epigenetic regulation can also occur in the form of transcriptional machinery interaction with ncRNAs including microRNAs (miRNAs), long noncoding RNAs (lncRNAs), and circular RNAs (circRNAs). Emerging evidence suggests that there is a relationship between different ncRNAs and their roles in the regulation of gene networks involved in the development of metabolic diseases including obesity and NAFLD [18,42,43,44,45]. The best characterized category of ncRNA species is the miRNAs class. Mounting evidence revealed that dysregulation in miRNAs expression is associated with molecular processes of various metabolic and pathophysiologic liver diseases, including NAFLD conditions [46,47]. Indeed, several differentially expressed miRNAs were associated with the pathogenesis of NAFLD and its subtype NASH, both in humans and in experimental models [48,49,50]. Moreover, plasma miRNA expression signatures could serve as a biomarker to differentiate between several types of liver injury such as simple steatosis, NASH, fibrosis and, ultimately, HCC [51,52]. Hence, the ncRNAs new emerging field of research is expected to significantly increase our understanding of the fundamental epigenetic mechanisms that contribute to NAFLD with the hope of developing potential biomarkers for diagnosis, prognosis, and treatment of the disease. Next, we focus on discussing the currently available knowledge regarding the best characterized epigenetic changes, such as DNA methylation and histone modifications, and how these alterations contribute to the development and progression of NAFLD in response to nutritional intake. As for advancement in the understanding of the mechanistic roles of ncRNAs in NAFLD, we refer the readers to recent well-detailed reviews [18,53,54,55].

### 2.1. DNA Methylation and NAFLD

DNA methylation is one of the most characterized biological process of the epigenome. This mechanism typically refers to the addition of a methyl group on a cytosine (C) with guanine (G) as the next nucleotide on DNA, known as CpG sites. CpG sites, usually referred to as CpG islands, commonly present with higher frequency at the promoter regions of the genes than that of other sites [34]. In the human genome, 70–80% of the 28 million CpG dinucleotides are methylated [56]. Interestingly, these dynamic CpGs cohabit with gene regulatory elements, particularly enhancers and transcription-factor-binding sites. The methylation is processed by a family of enzymes; the DNA methyl transferases (DNMTs) [57], which use S-adenosylmethionine (SAM) generated by one-carbon metabolism. Hypermethylation of CpG islands usually results in gene transcription silencing [58], while hypomethylation of promoters may activate gene transcription. Increasing numbers of studies indicate that DNA methylation patterns are susceptible to specific change in response to cellular and tissue microenvironments [59] and contribute to the epigenetic networks that operate to turn genes on and off in response to various signals [60]. More importantly, alterations in DNA methylation patterns can take place during aging and in pathologic states, such as metabolic diseases [61].

DNA methylation is one of the keys to how environmental conditions, particularly diet and nutritional status, modulate gene expression at the transcriptional level. Because DNA methylation relies on the availability of S-adenosylmethionine, which is synthetized from several nutrients, diet is one of the strongest factors that affects DNA methylation pathways [62]. For example, western type diet, which is known to promote obesity, also alters DNA methylation [63], thereby changing the expression of several genes involved in lipid metabolism [64]. DNA methylation patterns induced by dietary fatty acids are specifically linked with dysfunctions in cellular lipid metabolism and fatty acid oxidation [62,65]. However, much less is known about DNA methylation in NAFLD, and much more needs to be done. With recent progress in epigenetic tools such as high-throughput sequencing and methylation arrays, attempts were made to detect methylation signals and uncover the regulation of DNA methylation by HFD and its role in tissue-specific transcription control in NAFLD. In this context, relevant animal and human studies related to these aspects will be discussed next.

*Data from animal studies:* evidence of altered hepatic genomic DNA methylation in NAFLD is demonstrated by several animal studies; in particular, rodents. Maternal HFD was shown to alter littermates’ DNA methylation, as well as to favor the development of NASH and hepatic fibrosis [66]. Rat offspring born from mothers fed HFD during pregnancy and lactation periods develop the NAFLD phenotype, as well as changes in cyclin dependent kinase inhibitor 1A *(*Cdkn1a) gene expression and corresponding DNA methylation levels [67]. Methionine is an essential amino acid that plays major roles through its metabolites, which regulates a number of cellular functions. Mounting evidence from animal studies suggests that methyl-group donors including folate, betaine, and choline can alter DNA methylation patterns [68,69]. Further evidence in support of the importance of DNA methylation in NAFLD comes from studies in mice showing that dietary restriction of methyl donors or impairment of methyl donor metabolism alters DNA methylation and promotes NAFLD and liver injury [70]. By contrast, dietary methyl donor supplementation appears to protect rodents from high-fat/sucrose diet-induced hepatic steatosis [71]. Betaine was also found to relieve HFD-induced fatty liver in association with modification of DNA methylation [72].

In a mouse model of high-fat-sucrose-induced hepatic steatosis, supplementation with methyl donors containing folic acid, choline, betaine, and Vitamin B12 improved liver steatosis by reversing the methylation status in several genes including the sterol regulatory element binding transcription factor 2 (*Srebf2*) [68,73]. In another animal study, folate was reported to affect the expression of genes regulating fatty acid synthesis, and folate deficiency-induced TG accumulation in the liver [74]. Wang et al. [75] showed that betaine supplementation decreased DNA methylation of the microsomal triglyceride transfer protein (*Mttp)* gene promoter in mice and induced global methylation over the genome compared to HFD. These changes of DNA methylation induced by betaine supplementation promoted hepatic TG export and attenuated liver steatosis in mice fed HFD. Several studies also showed that epigenetic changes can regulate the transcription factor peroxisome proliferator-activated receptor γ (PPARγ), which is known as a master regulator of lipogenic genes involved in fatty liver diseases. PPARγ overexpression in the liver induced by HFD feeding or pathophysiological stresses leads to lipid accumulation, and consequently, development of NAFLD. Blocking *Pparγ* gene expression in the liver of HFD-fed mice reduced not only lipid accumulation, but also the expression of inflammatory genes, which is an indication of NASH progression [76]. In agreement with these studies, we recently reported that both HFD and palmitic acid alter global and *Pparγ* promoter DNA methylation leading to a significant induction of PPARγ expression and enhanced lipid retention in the liver, which lead to NAFLD development [38].

The nuclear factor-erythroid 2-related factor-2 (NRF2) is another transcription factor known to play a pivotal role in liver diseases [77]. Resveratrol attenuated hepatic DNA methylation at the *Nrf2* promoter region in mice fed an HFD, and this effect was correlated with a reduction in TG levels and expression of lipogenesis-related genes [78]. Thus, NRF2 signaling pathways could be a potential target to develop a preventive and therapeutic strategy to reduce NAFLD. Furthermore, several potential genes coding for enzymes involved in NAFLD were also reported to be susceptible to methylation and contribute to altered hepatic metabolism and cellular transformation. Glycine N-methyltransferase (GNMT) is the most important enzyme regulating S-adenosyl-L-methionine metabolism that is frequently decreased in liver disease, including NAFLD, cirrhosis, and HCC [79]. Likewise, Borowa–Mazgaj et al. reported that the development of NAFLD and NAFLD-derived HCC was characterized by decreased *Gnmt* gene expression and this was mediated by gradual DNA methylation in the promoter region in *Gnmt* [80].

Based on this knowledge, animal studies are beginning to examine therapeutic values of certain pathways for the treatment of NAFLD. For example, a recent research work showed that therapeutic targeting of hepatic methylation-controlled J protein (MCJ) with nanoparticle- and GalNAc-formulated siRNA efficiently prevented liver lipid accumulation and fibrosis in multiple NASH mouse models [81]. However, although studies in rodents provided crucial insights into the NAFLD onset and progression, the translatability between animals and humans should be carefully considered.

*Data from human studies:* differential DNA methylation was also associated with the pathogenesis of NAFLD in human. DNA methylation signatures of liver biopsies collected from patients with NAFLD revealed broad changes in the methylation profile compared to that of healthy individuals [82,83,84,85,86,87]. Growing evidence indicates that hepatic DNA methylation and insulin resistance in NAFLD patients are critical factors for the progression of the disease from simple steatosis to severe fibrotic NASH [88]. Indeed, hepatic DNMT levels were found to be increased in patients with steatohepatitis versus those with simple steatosis [86]. Hardy et al. found that the plasma methylation of PPARγ positively correlates with the severity of NAFLD [89]. In a case-control study of NAFLD patients, hepatic DNA promoter methylation in PPARγ coactivator 1- α (*PGC1-α*), was significantly associated with differential liver DNA methylation in NAFLD and peripheral insulin resistance [85]. Moreover, the methylation level of *Pparγ* was found to be positively correlated with liver fibrosis levels in rat models as well as in NAFLD patients [90]. Thus, circulating PPARγ DNA could be used as a potential biomarker for stratification of liver fibrosis in nonalcoholic fatty liver disease [89]. In agreement with this suggestion, another study carried out on individuals diagnosed with NAFLD indicated that DNA methylation at specific CpGs within *Pparα*, *Pparγ*, *TGFβ1*, *Collagen 1A1*, and *PDGFα* genes can distinguish mild from severe NAFLD-associated fibrosis [83].

Furthermore, Ahrens and colleagues identified an association between increased methylation at a CpG site (cg11669516) in the first intron and reduced expression of insulin-like growth factor binding protein 2 (*igfbp2*) gene in NAFLD and NASH patients [82]. These results are also supported by a recent cohort study indicating that IGFBP2 levels are inversely associated with the risk of NAFLD [91]. Similarly, Fahlbusch et al. demonstrated that circulating levels of IGFBP2 are lower in patients with NAFLD and NASH, and are restored after weight loss following bariatric surgery along with reductions in hepatic fat content [92]. A recent epigenomic study suggested that differentially methylated genes might distinguish patients with advanced NASH from simple steatosis [93]. There is evidence showing that mitochondrial gene NADH dehydrogenase 6 gene (*MT-ND6*), which was transcriptionally silenced by promoter hypermethylation, was significantly associated with the histological severity of NAFLD [86].

Based on all these data and those summarized in (Table 1), methylation status might be used as a parameter to improve the diagnosis of NAFLD and to differentiate between disease subtypes. However, the mechanisms by which HFD exerts its specific effects on epigenetic landmarks and DNA methylation, which could enhance lipid accumulation in hepatocytes promoting NAFLD, are only beginning to surface. Therefore, it merits more systematic studies to provide more unequivocal findings and research in the field of cell-free DNA that reflects gene methylation status in the liver. This would be a potential noninvasive biomarker of liver damage, as it was suggested by Hardy et al. [89].

### 2.2. Histone Post-Translational Modifications in NAFLD

Histone modifications were identified as another epigenetic determinant of chromatin structure and gene expression. Changes include mainly histone acetylation, methylation, phosphorylation, ribosylation, ubiquitination, and sumoylation. Among these, acetylation/deacetylation and methylation/demethylation mechanisms were the most studied modifications over the past decade. These epigenetic processes, which occur in response to various conditions including diets, are characterized by dynamic changes of aminoacidic residues in the histone tails [95]. A series of enzymes including histone acetyltransferase (HAT), histone deacetylase (HDAC) [96,97], and methyltransferase are accountable for ‘writing’ or ‘erasing’ the epigenetic modifications. Alterations in the activity and/or levels of any of these enzymes may impact chromatin structure and subsequent gene expression. Moreover, abnormal histone modifications contribute to metabolic disorders and consequently fatty liver disease [98]. Hence, a precise understanding of this epigenetic process may provide new perspectives in the discovery of novel epigenetic targets, which may provide important leads to design future functional studies and potential epigenetic-targeting drugs for NAFLD.

*Histone acetylation:* altered expression and activity of HAT modifying enzymes were reported to influence gene expression profiles in NAFLD, leading to aberrant hepatic metabolism and cellular transformation [88]. More recent research revealed that the dysfunction of lysine acetylation is involved in NAFLD and other metabolic diseases, including obesity, cardiovascular disease, hypertension, and T2DM [99]. For example, a study indicated that blocking the hyperacetylation of lysine 9 and 36 at histone 3 (H3K9 and H3K36) in the promoter of *SREBP1c, FASN,* and ATP citrate lyase (*ACLYS*) genes prevented the development of NAFLD (Table 2) [100]. In addition, a genome-wide analysis of histone 3 at lysine 9 acetylation (H3K9ac) in the liver of mice fed control or HFD demonstrated that approximately 17% of the differentially expressed genes were associated with changes in H3K9ac in their promoters [101]. In agreement with this, another study used HFD-fed mice to illustrate that hepatic lipid accumulation caused aberrant histone H3K4 and H3K9 trimethylation in *Pparα* and other genes involved in lipid metabolism, which may contribute to the pathogenesis of NAFLD [102].

Little is known regarding the role of HATs in the development of NAFLD. The p300 protein, a histone acetyltransferase HAT family member, is an important element of the transcriptional machinery that contributes in the regulation of chromatin structure and transcription initiation. A previous study indicated that p300 upregulation results in NAFLD, insulin resistance, and inflammation [103]. Glucose-activated p300 acetylated Lys-672 of the carbohydrate-responsive element-binding protein (ChREBP) and increased its transcriptional activity, leading to increased hepatic lipogenesis and the development of NAFLD [88,103]. In a recent study, Chung et al. identified tannic acid as a novel histone acetyltransferase inhibitor preventing NAFLD [100]. Therefore, suppression of hepatic p300 activity may be useful target for the treatment of hepatic steatosis and pharmacological p300 blockers may represent a potential option for NAFLD treatment.

*Histone deacetylation:* Several HDACs were reported to play a role in the pathogenesis of NAFLD. Sirtuin type 1 (SIRT1), which belongs to the silent information regulator-2 family, is the most studied member of the class III histone deacetylases [106]. SIRT1 is an important regulator of lipid and carbohydrate metabolism. Through its deacetylation capacity, SIRT1 was also shown to play a role in the pathophysiology of NAFLD and metabolic diseases. In this respect, Colak et al. reported that the deacetylation of SIRT1 is responsible for the regulation of several proteins involved in the pathogenesis of NAFLD [107]. For instance, SIRT1 was shown to potentiate fatty acid oxidation, mitochondrial biogenesis and turnover through deacetylation of its targets such as PGC-1α [108]. In response to caloric restriction, SIRT1 activates PGC-1α by deacetylation of lysine residues, thereby enhancing mitochondrial function [109]. The deacetylation effect of SIRT1 on histone was reported to improve hepatic steatosis [110]. In support to these findings, hepatocyte-specific deletion of Sirt1 resulted in hepatic steatosis and inflammation [111], whereas both transgenic SIRT1 mice and overexpression of SIRT1 specifically in the liver showed lower hepatic steatosis along with better glucose tolerance [112]. Another study revealed that SIRT1 levels were significantly reduced in a rodent model of HFD-induced NAFLD [113] as well as obese patients with severe steatosis [114]. SIRT1 transgenic mice exposed to a HFD showed a dramatic resistance to the development of HCC and damage in hepatocytes triggered by a chemical carcinogen [115]. Furthermore, Luo et al. reported that docosahexaenoic acid (DHA; C22; n-3) improved NAFLD by activating Sirt1 in a high-fat diet-induced NAFLD mouse model and prevented the accumulation of palmitic acid-induced lipid droplets, the decrease of fatty acid oxidation and the reduction of SIRT1 level in HepG2 cells [116]. Collectively, these data indicate that SIRT plays an important role in epigenome and metabolome in association with NAFDL development.

Several other HDACs are known to play a crucial role in NAFLD. Histone deacetylase 3 (HDAC3) was shown to be essential for the maintenance of chromatin structure and its liver-specific deletion caused both advanced fibrotic NAFLD and HCC [117]. A further study also demonstrated that histone HDAC3 to be a key epigenomic coregulator in liver, and that hepatic suppression of HDAC3 in liver results in remarkable steatosis [118]. Defects in the regulation of circadian clock genes by HDAC3 may lead to abnormal lipid metabolism in the liver, which may increase the risk of NAFLD [119]. HDAC8 is another histone deacetylase, commonly upregulated in dietary and genetic obesity promoted HCC mouse models as well as in human HCC cells and tissues [120]. HDAC8 promoted insulin resistance as well as cell proliferation, while its suppression induced insulin sensitivity and inhibited tumorigenesis in HCC [120].

*Histone methylation:* several studies reported that NAFLD development and progression are associated with alterations in the pattern of histone methylation profiles. Histone methylation marks are responsible for the epigenetic regulation of chromatin structure through addition or removal of methyl groups from lysine residues of histone tails [121]. Histone methylation is mediated by histone methyltransferases (HMTs) [122]. Methylation marks and their respective demethylation of lysine residues within histones H3 and H4 act as epigenetic switches that can either activate or repress gene expression [121]. Kim et al. reported that the histone H3 lysine 4 (H3K4) methyltransferase MLL4/KMT2D directs overnutrition-induced steatosis via its function as coactivator for PPARγ2 (Table 2) [104]. Additional investigations suggested that H3K4 an H3K9 trimethylation may contribute to hepatic steatosis and disease progression [102]. Indeed, this group of researchers has shown that hepatic lipid accumulation is linked with aberrant histone H3K4 and H3K9 trimethylation in *PPARα* and increased expression of genes involved in lipid metabolism in HFD-fed mice [114]. The methylation transferase suppressor of variegation 3-9 homologue 2 (Suv39h2) is significantly elevated in diet-induced obese mice and NAFLD patients and represses both *Sirt1*and *Pparγ* genes expression [94]. Another histone methyltransferase Enhancer of Zeste Homolog 2 (EZH2), which catalyzes trimethylation of H3K27 (H3K27me3) for transcriptional repression, was shown to play a key role in liver diseases. The reduction of EZH2 expression in the liver of NAFLD rats and fatty acid-treated hepatocytes is inversely correlated with lipid accumulation and inflammatory marker expression [123]. In agreement with this, inhibition of EZH2 recapitulated the steatosis-related phenotypes.

Histone demethylation: histone demethylation is carried out by histone demethylases (HDMs), which remove methyl groups from modified histones, thereby activate or repress gene transcription. Several histone demethylases were identified and classified into two classes: FAD-dependent amine oxidases (LSD demethylases) and Fe(II)- and α-ketoglutarate-dependent Jumonji C (JmjC) domain-containing demethylase (JMJD demethylase) [124]. MJD1C of Jumonji family was identified as a critical epigenetic factor for lipogenesis. Suppression of JMJD1C in animal models can protect from dietary-induced NAFLD, while its overexpression promotes lipogenesis to increase hepatic and plasma triglyceride levels [125]. Histone H3K9 demethylase JMJD2B is a member of the JMJD2 family. JMJD2B specifically catalyzes the removal of di- and trimethylated H3K9 (H3K9me2/me3), converting both histone marks to the monomethylated state. JMJD2B was shown to play a role in the development of hepatic steatosis through upregulation of PPARγ2 and steatosis target genes including CD36 and fatty acid-binding protein [105]. Not long ago, Kim et al. provided evidence that JMJD2B induces LXRα-dependent lipogenesis by removing repressive histone marks H3K9me2 and H3K9me3 near LXREs of lipogenic gene promoters leading to the development of NAFLD [126]. Moreover, an additional H3K9 HDM, Plant homeodomain finger protein 2 (Phf2), was shown to erase H3K9me2 methyl-marks on the promoter of carbohydrate-responsive element-binding protein, (ChREBP) thereby protects liver from the pathogenesis progression of NAFLD [127]. Together, these findings suggest that the understanding of histone epigenetic dynamic changes underlying the development of NAFLD may deliver new insights into the physiopathology of the disease enabling the development of novel therapeutic and prevention modalities.

## 3. Epigenetic Studies’ Limitations

Existing preclinical and clinical studies are providing evidence that epigenetic mechanisms such as DNA methylation and histone modifications play crucial roles in several metabolic diseases including NAFLD. In fact, epigenetic processes bridge the genetic and environmental factors such as diet, which contribute to transcriptional and posttranslational control of gene expression and consequently influence NAFLD and its more advanced clinical phenotype. In this respect, epigenetic modifications could have future application as effective diagnostic and therapeutic tools for NAFLD. However, many aspects of NAFLD biology remain enigmatic, and the research area still has important limitations: (i) since epigenetics applied to metabolic diseases is a relatively new field of investigation, a significant limitation is the current preclinical models of NAFLD, which make it difficult to study the interplay between diet and epigenetic changes. Although current animal models are necessary systems for biologically characterizing the disease, there is no consensus on a suitable animal model that could represent both the pathophysiology and histopathology in human NAFLD. In fact, most of the existing animal systems represent only a specific aspect of the disease rather than the whole spectrum. Moreover, diet used in animal studies to introduce NAFLD phenotype does not reflect the depth of dietary variation in humans, which clearly highlights the need to move to clinical studies. There are also limitations with respect to human studies. These include difficulties in capturing with accuracy an individual’s response to complex environments. For example, the response that we can measure in one set of conditions may not be apparent in another set of circumstances. In addition, epigenetic processes are complex and depend on a variety of parameters including sex, stress, genetic variants, and tissue specificity. Besides, human studies are technically difficult due to the invasive procedure to get biopsies from the liver. Alternatives such as the development of new methods for quantification of DNA methylation from circulating cell-free DNA isolated from patient plasma has the potential to overcome this limitation [128]. With such an approach, epigenetics biomarkers are becoming close to clinical reality, as demonstrated by the example of the circulating, cell-free, DNA-based epigenetic biomarker methylated Septin9, which shows great promise as a tool to diagnose HCC in patients with cirrhosis [129]. (ii) Further to this difficulty is that certain dietary patterns are known to cause metabolic disorders mediated by epigenetic alterations but, in which metabolic tissue, by which mechanism, and in which physiological and pathological conditions? All remain to be determined. Understanding the dynamic relationship between food consumption, epigenetic changes, and genome may provide insight into how to target molecules involved in NAFLD either from a nutritional perspective or an epigenetic standpoint. (iii) Studies on DNA methylation patterns associated with diet quality in larger sample size racially diverse research cohorts are lacking. In this respect, preliminary data provided key evidence that higher diet quality has a beneficial effect on the lifespan, and adopting a healthy diet is crucial for maintaining healthy aging [130]. Moreover, Ma et al. reported that the whole blood DNA methylation signatures of diet were associated with cardiovascular disease risk factors and all-cause mortality [131]. Therefore, further studies are required to measure accurately DNA methylation after exposure to various diet as this may help understanding individual differences in responses to diet and diet-related chronic disease. (iv) While most of the studies discussed above proved that epigenetic alterations can influence gene expression in NAFLD when considering each epigenetic mechanism separately, research that combines all epigenetic layers in a studied model is missing. The identified and yet to be identified epigenetic mechanisms may interact and overlap among all and with several cellular factors, such those involved in the transcriptional machinery, to modulate target gene expression. Other issues to take in consideration are unknown molecular pathways from other primary metabolic tissues, along with secreted molecules involved in NAFLD that are also subject to the same epigenetics changes and may affect the diseases outcome. All these complicate attempts to identify the primary epigenetic trigger of aberrant gene expression and pathological role of an epigenetic event in a single organ. Hence, future studies need to exploit the whole-epigenome in greater depth and breadth than previously possible. Such effort may permit the screening and analysis of DNA methylation, modified histone landscapes, and ncRNAs regulation all in a single sample, as well as in different tissues of an organism. This approach would possibly help determine and understand interdependencies in the epigenetic landscape and its link to genome function under various inputs. (v) Due to the advances in the chemogenetic RNA-labeling and next-generation sequencing, several cellular RNAs were also found to dynamically and reversibly undergo different chemical modifications post-transcriptionally, a process called epitranscriptome. The dynamic and reversible modifications of RNA were found in multiple classes, such as mRNA, rRNA, tRNA, and noncoding RNA, with increasing evidence suggesting that they play important roles in post-transcriptional gene regulation. Indeed, the most abundant internal mRNA modification in eukaryotic cells that provides a new perspective for the regulation of gene expression and exhibit essential roles in physiological processes including hepatic functions and various liver diseases is N6-methyladenosine (m^6^A) [132]. In fact, recent studies investigated the role of m^6^A RNA methylation in disorders of hepatic lipid metabolism, showing that hyper-methylated m^6^A sites in HFD-induced fatty livers are enriched for lipid-associated pathway processes, while hypo-methylated m^6^A sites are associated with translation-associated processes [133]. Nevertheless, important, m^6^A mechanisms remain unexplored in the context of NAFLD. Thus, comprehensive studies to demonstrate a potential linkage between diet, genetics, epigenetics, and epitranscriptomic regulation of gene expression would offer potential new insights for the understanding the different stages of NAFLD.

## 4. Conclusions

Although associations between epigenetic modifications and NAFLD was demonstrated, it is still not clear whether epigenetic alterations lead to NAFLD or rather the onset of NAFLD is the trigger for different alterations of epigenetic landscape. In this regard, further mechanistic studies are becoming a real necessity to better dissect causal from correlative relationships in the field. Additionally, more fine-tuned research needs to be achieved towards understanding how in an individual, diet, epigenetic layers, and genetic make-up crosstalk to alter hepatic gene expression, leading to the pathogenesis NAFLD. Finally, combining the latter research with the implementation of dietary interventions such as caloric restriction, Mediterranean diet, intermittent fasting, and their ability to reverse disease state, epigenetics would allow the design of a modular switch ‘on/off’ that controls gene expression in response to a specific diet to reverse metabolic diseases such as obesity, metabolic syndrome, and NAFLD.

## Figures and Tables

**Table 1:** Relevant DNA methylation alterations associated with gene expression in nonalcoholic fatty liver disease.

Gene	Stage	Associated Disease Mechanisms	References
*Srebf2*	Hepatic steatosis	Supplementation with methyl donors containing folic acid, choline, betaine, and Vitamin B12 improved liver steatosis by reversing the methylation status in the promoter region of sterol regulatory element binding transcription factor 2 (*Srebf2*)	[68,73]
*Mttp*	Hepatic steatosis	Betaine supplementation decreased DNA methylation of the microsomal triglyceride transfer protein (*Mttp*) gene promoter in mice and improved HFD-induced hepatic steatosis	[75]
*Pparγ*	NAFLD	HFD and palmitic acid alter *Pparγ* promoter DNA methylation leading to a significant induction of PPARγ expression and enhanced fat accumulation in mice liver, which may lead to NAFLD	[38]
*Nrf2*	NAFLD	Treatment of HepG2 cells with high glucose enhanced methylation level of the Nrf2 promoter whereas Resveratrol reversed the effect, which led to a reduction in TG levels and the expression of lipogenesis-related genes	[78]
*Gnmt*	HCC	Reduced *Gnmt* expression caused by promoter cytosine DNA hypermethylation is one of the key molecular events in the development of NAFLD-derived HCC	[80]
*Pparγ*	NAFLD	Hypermethylation at the *Pparγ* promoter of plasma DNA correlated with with fibrosis severity in patients with NAFLD	[89]
*PGC1-α*	NAFLD	Hepatic DNA methylation of of PPARγ coactivator 1- α (*PGC1-α)* promoter significantly correlates with peripheral insulin resistance and is associated with decreased *PGC1-α* mRNA expression	[85]
*Pparα, Pparγ, TGFβ1, Collagen 1A1,* *PDGFα*	NAFLDfibrosis	DNA methylation at specific CpGs within *PPARα, PPARγ, TGFβ1, Collagen 1A1,* and *PDGFα* genes can distinguish mild from severe fibrosis in NAFLD patients	[83]
*IGFBP2*	NASH	The IGFBP2 (insulin-like growth factor binding protein 2) locuswas hypermethylated and its mRNA downregulated in NASH	[82]
*MT-ND6*	NAFLD	Hepatic methylation and transcriptional activity of the *MT-ND6* gene are significantly associated with the histological severity of NAFLD	[86]
*Sirt1, Pparγ*	NAFLD	Suv39h2 is significantly elevated in diet-induced obese mice and NAFLD patients, and it increases the methylation levels at histone H3 lysine 9 of both *Sirt1* and *Pparγ* to suppress the gene expression	[94]

*COL1A1,* Collagen type I α1; *Gnmt,* Glycine N-methyltransferase; HFD, high-fat diet; IGFBP2, insulin-like growth factor binding protein 2; *MT-ND6*, mitochondrial gene NADH dehydrogenase 6; NAFLD, nonalcoholic fatty liver disease; NASH, nonalcoholic steatohepatitis; *Nrf2,* Nuclear factor-erythroid 2-related factor-2; *PDGFα*, Platelet-derived growth factor alpha; *PGC1-α,* PPARγ coactivator 1- α; PPARα, peroxisome proliferator-activated receptor α; PPARγ, peroxisome proliferator-activated receptor γ; *Srebf2*, sterol regulatory element binding transcription factor 2; Suv39h2, the methylation transferase suppressor of variegation 3-9 homologue 2; *TGFβ1,* transforming growth.

**Table 2:** Examples of histone modifications and their association with aberrant gene expression in nonalcoholic fatty liver disease.

Gene	Stage	Association between Epigenetic Determinant and Gene Expression	References
*SREBP1c, FASN ACLYS, Pparγ*	NAFLD	Blocking the hyperacetylation of lysine 9 and 36 at histone 3 (H3K9 and H3K36) in the promoter of lipogenesis-related genes (*SREBP1c, FASN, ACLYS,* *Pparγ*) prevented NAFLD	[100]
*Pparα*	NAFLDSteatosis	Hepatic lipid accumulation induced aberrant H3K9me3 and H3K4me3 status in *Pparα* gene and other hepatic lipid catabolism network genes, which may contribute to hepatic steatosis and the pathogenesis of NAFLD	[102]
*ChREBP*	Hepatic Steatosis	p300 associates and regulates carbohydrate-responsive element–binding protein (ChREBP) transcriptional activity by acetylation. Inhibition of hepatic p300 activity may be beneficial for treating hepatic steatosis	[103]
*Pparγ2*	Hepatic steatosis	Histone H3 lysine 4 (H3K4) methyltransferase MLL4/KMT2D directs overnutrition-induced murine steatosis via its coactivator function for PPARγ2	[104]
*Pparγ2, CD36*, *FABP4, PLIN2, CIDEC,*	Hepatic steatosis	Overexpressing JMJD2B upregulated *Pparγ2* expression which lead to a concomitant increase in its steatosis target genes by removing repressive histone marks H3K9me2 and H3K9me3 near LXREs of lipogenic gene promoters leading to the development of NAFLD	[105]

ACLY, ATP-citrate lyase; CD36, fatty acid translocase; ChREBP, carbohydrate-responsive element–binding protein; FABP4, fatty acid-binding protein 4; FASN, fatty acid synthase; HFD, high-fat diet; JMJD2B, jumonji domain-containing protein 2B histone demethylase; LXRα, liver X receptor α; NAFLD, nonalcoholic fatty liver disease; NASH, non-alcoholic steatohepatitis; p300, transcriptional coactivator with histone acetylase activity; PPARγ, peroxisome proliferator-activated receptor gamma; SIRT1, Sirtuin type 1;SREBP-1c, sterol regulatory element-binding protein 1c.

## Data Availability

Not applicable.

## Abbreviations

Arnlt	Aryl hydrocarbon receptor nuclear translocator-like
CpG	Cytosine-phospho-guanine
CXCL5	C-X-C Motif Chemokine Ligand 5
DNMT	DNA methyltransferase
HATs	Histone acetyltransferases
HCC	Hepatocellular carcinoma
HDACis	Histone deacetylase inhibitors
HDACs	Histone deacetylases
HDMs	Histone demethylases
HFD	High-fat diet
HMTs	Histone methyltransferases
HSC	Hepatic stellate cell
IR	Insulin resistance
MetS	Metabolic syndrome
mTOR	mammalian target of rapamycin
NAFLD	Nonalcoholic fatty liver disease
NASH	Nonalcoholic steatohepatitis
PPARγ	Peroxisome proliferator-activated receptor γ
PTEN	Phosphatase and tensin homolog
S6K1	Ribosomal protein S6 kinase beta-1
SAM	S-adenosyl methionine
Sirt1	Sirtuin 1
STAT5	Signal transducer and activator of transcription 5
T2DM Type 2	diabetes mellitus
TG	Triglyceride
UPS10	Ubiquitin-Specific Protease 10
α-SMA	Alpha Smooth Muscle Actin
